# Patient-reported outcome after patient-specific unicondylar knee arthroplasty for unicompartmental knee osteoarthritis

**DOI:** 10.1186/s12891-020-03776-3

**Published:** 2020-11-24

**Authors:** Viola Freigang, Markus Rupp, Christian Pfeifer, Michael Worlicek, Stefan Radke, Stephan Deckelmann, Volker Alt, Florian Baumann

**Affiliations:** 1 Department of Trauma Surgery, Regensburg University Medical Center, 93042 Regensburg, Germany; 2 Department of Orthopaedic Surgery, Rotkreuzklinikum Munich, Munich, Germany

**Keywords:** Unicompartmental knee arthroplasty (UKA), Outcome, Patient reported outcome measurement (PROM), Forgotten joint score (FJS)

## Abstract

**Background:**

Unicondylar knee arthroplasty was introduced in the late 1960s and remains a topic of controversial discussion. Patient-specific instruments and patient-specific implants are not yet the standard of care. The question remains whether this time-consuming and costly technique can be beneficial for the patient. The aim of this study was to evaluate whether a custom-made unicondylar knee arthroplasty leads to improved patient-reported outcome.

**Methods:**

This retrospective study evaluates the patient-reported outcome after custom-made unicondylar knee arthroplasty (CM-UKA, ConforMIS™ iUni® G2, ConforMIS Inc., Billerica, MA, USA). We evaluated 29 patients (31 knees) at an average of 2.4 years (range 1.2–3.6 years) after operation for unicondylar osteoarthritis of the knee. The target zone for the postoperative leg axis was a slight under-correction of 0–2° varus. Follow-up evaluation included the Forgotten Joint Score (FJS), the Knee Society Score (KSS), a Visual Analogue Scale (VAS) and a radiographic evaluation including a long-leg radiograph. Primary outcome measure was patient satisfaction based on the Forgotten Joint Score.

**Results:**

We found an excellent postoperative health-related quality of life with a mean FJS of 76.8 (SD 17.9) indicating a low level of joint awareness after CM-UKA. The mean preoperative KSS was 66.0 (SD 13.71) and 59.4 (17.9) for the KSS function score. The increase was 22.8 points for the KSS knee score (*p* < 0.0001) and 34.8 points for the KSS function score (*p* < 0.0001). The VAS for pain decreased from a mean of 5.4 (SD 1.8) to 1.1 (SD 1.2) (*p* < 0.0001). The malalignment rate with a postoperative deviation of more than 2° in the leg axis was 29%. There was no evidence of component loosening after a mean follow-up of 2.4 years.

**Conclusions:**

Custom-made unicondylar knee arthroplasty (CM-UKA) can provide improved clinical and functional outcomes for patients with isolated knee osteoarthritis of the medial compartment. We found excellent results regarding patient satisfaction and a low malalignment rate for CM-UKA. Further studies are needed to investigate long-term survivorship of the implant.

**Level of evidence:**

Level IV.

**Trial registration:**

Trial Registration number: Z-2014-0389-10 Regensburg Clinical Studies Center (REGCSC) 09/07/2014.

## Background

Although unicondylar knee arthroplasty (UKA) was already introduced in the late 1960s by Marmor et al. [1] it is still a topic of controversial discussion. Compared to total knee arthroplasty (TKA), implantation of UKA is less invasive due to a smaller skin incision, less blood loss and greater preservation of the patient’s anatomy [2–4]. Large, registry-based studies have shown better postoperative knee function and increased patient satisfaction but also increased revision rates in comparison to TKA [5, 6]. Recent studies on comparison of UKA and TKA proved a superior proprioceptive capacity and improved health-related quality of life (HR-QoL) for UKA [7]. A better understanding of the causes of failure has led to improved clinical results and higher patient satisfaction over the last decade [8]. In addition, prior studies have shown that surgeons who regularly perform implantation of UKA have a lower complication rate, especially lower mechanical complications [9, 10]. Introduction of patient-specific surgical instruments and implants are seen to have tremendous potential to improve the standard of care [11, 12]. In most studies, the term *patient-specific UKA* is associated with the use of patient-specific instruments for the implantation of a standard prosthesis. In this study, we used patient-specific instruments to implant a custom-made prosthesis based on a preoperative CT scan of the knee (ConforMIS™ iUni® G2, ConforMIS Inc., Billerica, MA, USA). Therefore, we use the term custom-made unicondylar knee arthroplasty (CM-UKA) to indicate that instruments and implants were patient-specific. Previous studies on patient-specific instrumentation of UKA have demonstrated superior implant fit compared to standard UKA [13]. The question remains whether this type of implant can be beneficial for the patient’s perception of the joint after arthroplasty. Conventional outcome measurement tools focus on functional aspects like the range of motion or ligamentous stability [14, 15]. In recent studies, more importance has been placed on patient-reported outcome. There is currently a lack of research into the impact of a patient-specific implant on patient-reported outcome (PRO). The purpose of this study is to investigate short-term PRO in patients with CM-UKA for unicompartmental osteoarthritis of the knee.

## Methods

### Study design

**Fig. 1:**
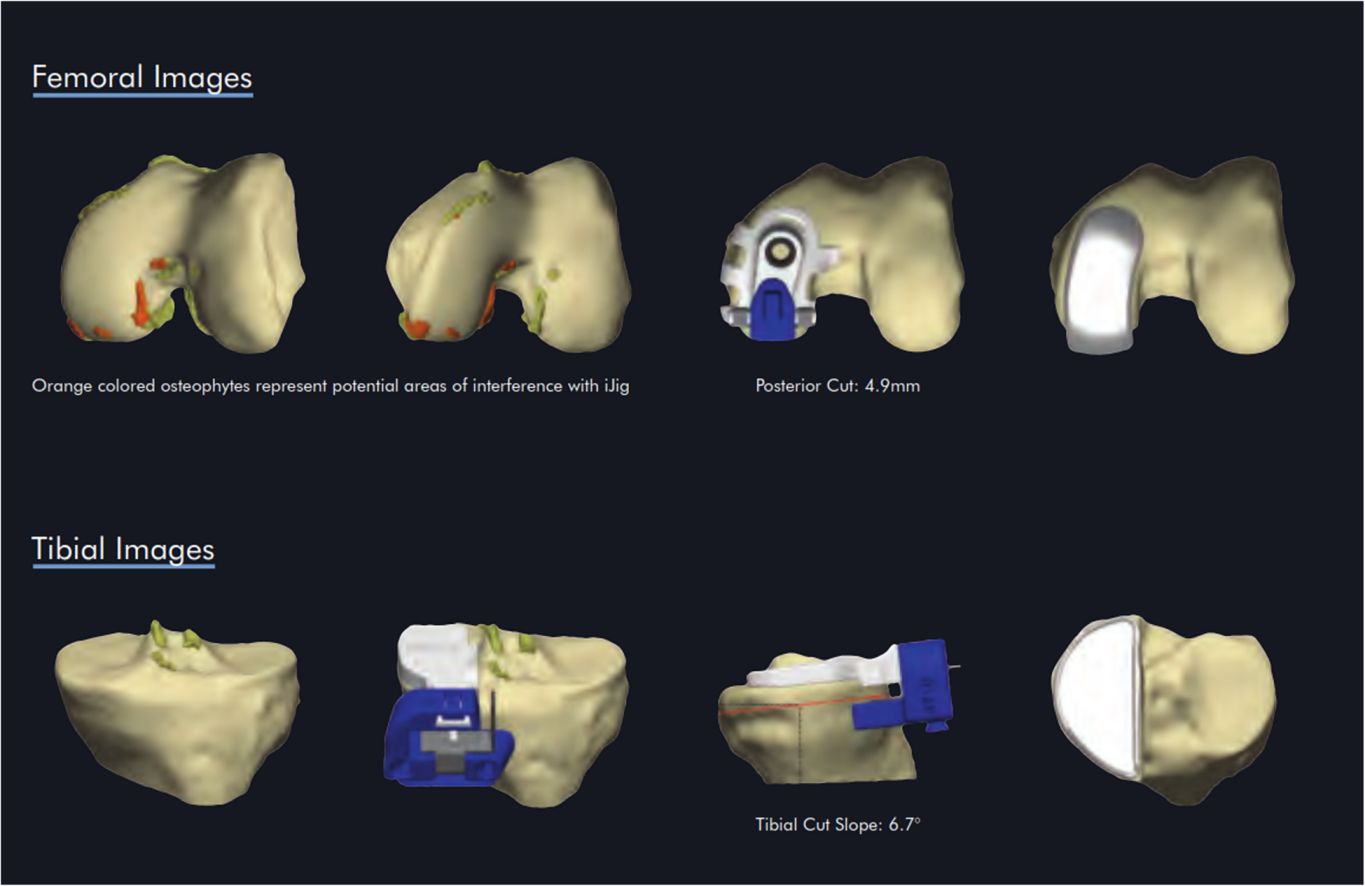
Preoperative planning procedure to determine optimal implant size and position

This retrospective, monocentric case study includes 31 knees in 29 consecutive patients who were treated with CM-UKA for medial unicompartmental osteoarthritis of the knee between January 2010 and December 2012. Our institution is a highly specialized unit with prospective data collection for all arthroplasty patients. We collected perioperative data of the study population from the hospital’s database retrospectively and performed clinical radiological re-evaluation at follow-up. The indication for CM-UKA was based on modified criteria by Kozinn and Scott [16]. The implants used were the second generation of the custom-made unicondylar arthroplasty implant (ConforMIS™ iUni® G2, ConforMIS Inc., Billerica, MA, USA). Preoperative planning and manufacturing process of the CM-UKA requires around 6 weeks. Based on a 3D reconstruction of a CT scan of the knee, the size and position of the patient-specific implant are determined (Fig. 1). To determine the leg axis, additional short distance scans of the hip and ankle joint have to be included in the CT scan. The planning software indicates osteophytes that have to be removed for correct positioning of the cutting guide instruments on femur and tibia. After removal of the osteophytes, the jig fits perfectly on the condyle. Placement of the saw guides is crucial to ensure correct transformation from the preoperative planning to definitive implant position. This type of implant has a fixed-bearing inlay and requires cementing of both components (femoral and tibial).

Inclusion criteria for this study were:

- isolated femoro-tibial OA of the medial compartment (grade II-IV according to Kellgren and Lawrence)
- sufficient bone stock in the medial compartment for anchoring of the implant
- intact cruciate ligaments and collateral ligaments

Exclusion criteria were:

- varus deformity greater than 15°
- osteonecrosis of the distal femur or proximal tibia
- post-traumatic or post-infectious OA
- lost to follow-up

Two patients were lost to follow-up, leaving 29 patients (31 UKAs) as study population. With a mean follow-up of 2.4 years (range 1.2–3.6 years), the remaining 31 CM-UKAs were evaluated clinically and radiographically by the first author. Primary outcome measure was the FJS at follow-up.

### Clinical evaluation

The average age of the sample was 67 years (range: 52–80 years) (Table 1). Twenty-three patients had prior surgery on the knee in their medical history (22 arthroscopies, 3 tibial osteotomies). All patients underwent conservative non-operative treatment prior to the arthroplasty (analgesia, anti-inflammatory drugs, injections, physical therapy, or acupuncture). Regarding the mechanical axis, the target zone was a slight under-correction of 0–2° varus [17, 18].

**Table 1:** Table showing patient demographics from our study

Age	67 years (52–80 years)
Sex	14 females (55%) 17 males (45%)
Compartment	31 medial (100%)
ASA^a^	7 patients with ASA II (23%) 24 patients with ASA III (77%)
BMI^a^	31.2 kg/m^2^ (24.6–45.4 kg/m^2^)
Follow-up	2.4 years (14 to 44 months)

^a^*ASA* American Society of Anesthesiologists, *BMI* Body Mass Index

The evaluation at follow-up was conducted by the first author and included medical history, a standardized clinical examination, and assessment of the following clinical scores:

- clinical examination including range of motion (ROM) and ligamentous stability
- Forgotten Joint Score (FJS) [19]
- Knee Society Score (KSS) [15, 20]
- Tegner Activity Level Scale [14]
- Visual Analogue Scale (VAS) for acute pain [21]
- Visual Analogue Scale (VAS) for patient satisfaction

### Forgotten joint score (FJS)

The FJS score is a self-administrated 12-item PRO tool measuring the loss of awareness of an artificial joint as an indicator for the postoperative outcome.

The score is reported on a scale from 0 to 100, where 100 represents the best outcome indicating that the patient is not aware of the artificial joint in everyday life. Validation studies proved excellent psychometric properties and revealed a minimum important clinical difference (MICD) of 14 points [19, 22–26].

The patients were asked to rate their level of satisfaction on a visual analog scale. The value was transformed into the following categories: 8–10 = “very satisfied”, 6–8 = “satisfied”, 4–6 = “neither satisfied nor dissatisfied”, 2–4 = “dissatisfied”, 0–2 = “very dissatisfied”.

### Radiological assessment

**Fig. 2:**
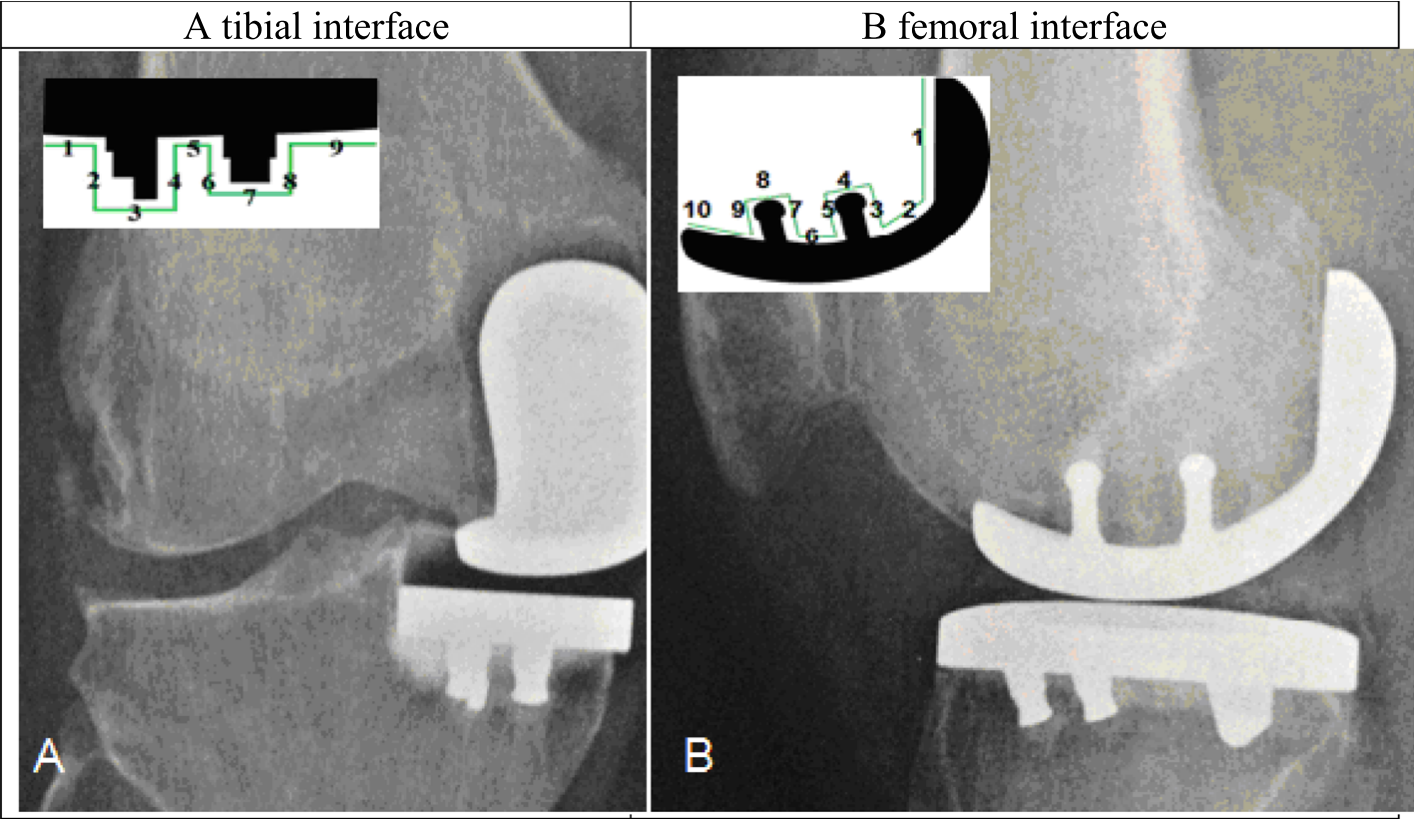
Radiographs with design of the specified regions for evaluation of presence and extent of radiolucency under the components

The radiographic analysis consisted of a weight-bearing long-leg view, an anterior-posterior view, a lateral view of the knee, and a patella axial view. The degree of OA was rated according to the Kellgren and Lawrence Score (KLS) [27] for pre- and postoperative images. The evaluation was made for each compartment separately. The retro-patellar OA was classified according to Sperner [28]. The radiological analysis was carried out using the zone method according to Gulati et al. [29] (Fig. 2. & Table 2). The thickness and location of radiolucent lines (RLL) was registered.

**Table 2:** Extend of radiolucent lines under the components

	Tibial interface (A)	Femoral interface (B)
none	*N* = 25; 80.6%	*N* = 30; 96.8%
1-2 mm	*N* = 4; 12.9%	*N* = 1; 3.2%
> 2 mm	N = 1; 3.2%	–

### Statistical analysis

Statistical analysis was performed using the software package SPSS (Version 23, SPSS Inc., Chicago, Illinois). With the limited number of cases presented in this study, assumption of normal distribution is critical. As a consequence, outcome data should be expressed as median. However, for comparison reasons, we present mean values ± standard deviation. The level of significance was at *p* < 0.05 for all tests.

Due to the fact that there is no previous data on joint awareness in CM-UKA, this preliminary study was designed as an exploratory pilot study without any a priori sample size calculation based on a primary endpoint. Since normal distribution could not be assumed for all variables (according to the Shapiro-Wilk normality test), a two-tailed Wilcoxon Signed Ranks Test was used to examine related samples; e.g. comparing pre- and post-operative results of the KSS, the VAS for pain, and the TAS. The Mann-Whitney U-Test was used to compare non-related samples.

The Ethics Committee at the University of Regensburg approved the study in May 2014 (Institutional Review Board Number 14–101-0135). The study was registered at the Center for Clinical Studies at the University Clinic of Regensburg at the 09/07/2014. Trial Registration number: Z-2014-0389-10- Regensburg Clinical Studies Center (REGCSC).

A written informed consent was obtained from all patients in accordance with the declaration of Helsinki.

## Results

### Clinical evaluation

Table 1 shows demographic data recorded in 31 knees. The average BMI was 31.2 (range, 24.6–45.4) and obesity was not a determining factor in whether or not to choose a UKA. The average pre-operative range of motion was 115° (range: 90–130). The mean preoperative KSS Knee score was 66.0 (SD 13.71) and 59.4 (17.9) for the KSS function score. The increase was 22.8 points for the KSS knee score (*p* < 0.0001) and 34.8 points for the KSS function score (*p* < 0.0001). No general complication had occurred by the time of follow-up (e.g. thromboembolism, hemorrhage, prolonged wound healing, or post-operative infection). At follow-up, 90.3% of patients reported either no pain or only occasional light pain. One patient was cane-dependent due to a gait disturbance as a consequence of a prior stroke. All other patients reported the ability to walk an unlimited distance. The mean FJS was 76.8 (SD 17.9) indicating a low level of joint awareness of the CM-UKA. The mean Tegner Activity Level improved from 2.6 (SD 0.9) preoperatively to 3.3 (SD 1.0) postoperatively (*p* < 0.0001). The VAS for pain during activity of daily living decreased from a mean of 5.4 (SD 1.8) to 1.1 (SD 1.2) (*p* < 0.0001). There was a significant improvement in all clinical scores that were measured at pre- and post-operatively (Table 3). 27 of the 31 patients were very satisfied (VAS 8–10), 2 satisfied (VAS 6–8), and 2 were not satisfied but not dissatisfied (VAS 4–6).

**Table 3:** Summary of outcome for the cohort. Clinical scores with means presented with standard deviation (SD) in the two groups; pre- and post-operatively with *p*-value

	Pre-operative: Mean (SD)	Post-operative: Mean (SD)	*P*-value
FJS	–	76.8 (17.9)	–
KSS Knee	66.0 (13.7)	88.8 (6.6)	< 0.001 *
KSS Function	59.4 (17.9)	94.2 (11.0)	< 0.001 *
Tegner	2.6 (0.9)	3.3 (1.0)	0.003 *
VAS-pain	5.4 (1.8)	1.1 (1.2)	< 0.001*
Sperner Score Patellofemoral joint	1.1 (0.6)	1.8 (0.8)	0.001 *
Kellgren Score Lateral compartment	0.3 (0.5)	1.45 (0.7)	< 0.001*
Femorotibial alignment	176° (2.3) -4° varus	179.4°(2.5) -0,6° varus	< 0.001 *

*level of significance α = 0.05 (Wilcoxon-Signed Rank Test)

### Radiological assessment

The short-term survival of the implant, considering revision and radiologic loosening to be failures, was 100%. There was no evidence of component loosening in any patient. Five patients showed partial RLL, of which one patient presented partial radiolucency in the femoral component in the very anterior region (zone 10) of less than 2 mm. Five patients showed partial lucency under the tibial component, only one of them > 2 mm. The very lateral zone (zone 1) was affected in 2 cases.

We recorded an increase of the mean KLS in the lateral compartment of 0.3 (SD 0.5) preoperatively to 1.5 (SD 0.7) at follow-up (*p* < 0.001). However, none of these patients complained of pain, joint effusion or other clinical symptoms from the lateral compartment degeneration. No patient with a BMI < 25 kg/m^2^ showed progression of OA in the lateral compartment.

The retro-patellar OA according to Sperner increased from a mean of 1,1 (SD 0,6) to 1.77 (SD 0.76) (*p* = 0.001). No patient with a BMI < 25 kg/m^2^ showed any sign of radiographic progression of the retro-patellar OA. No patient had any account of clinical symptoms or pain and the patellar femoral grind test [30] was negative in all patients.

**Fig. 3:**
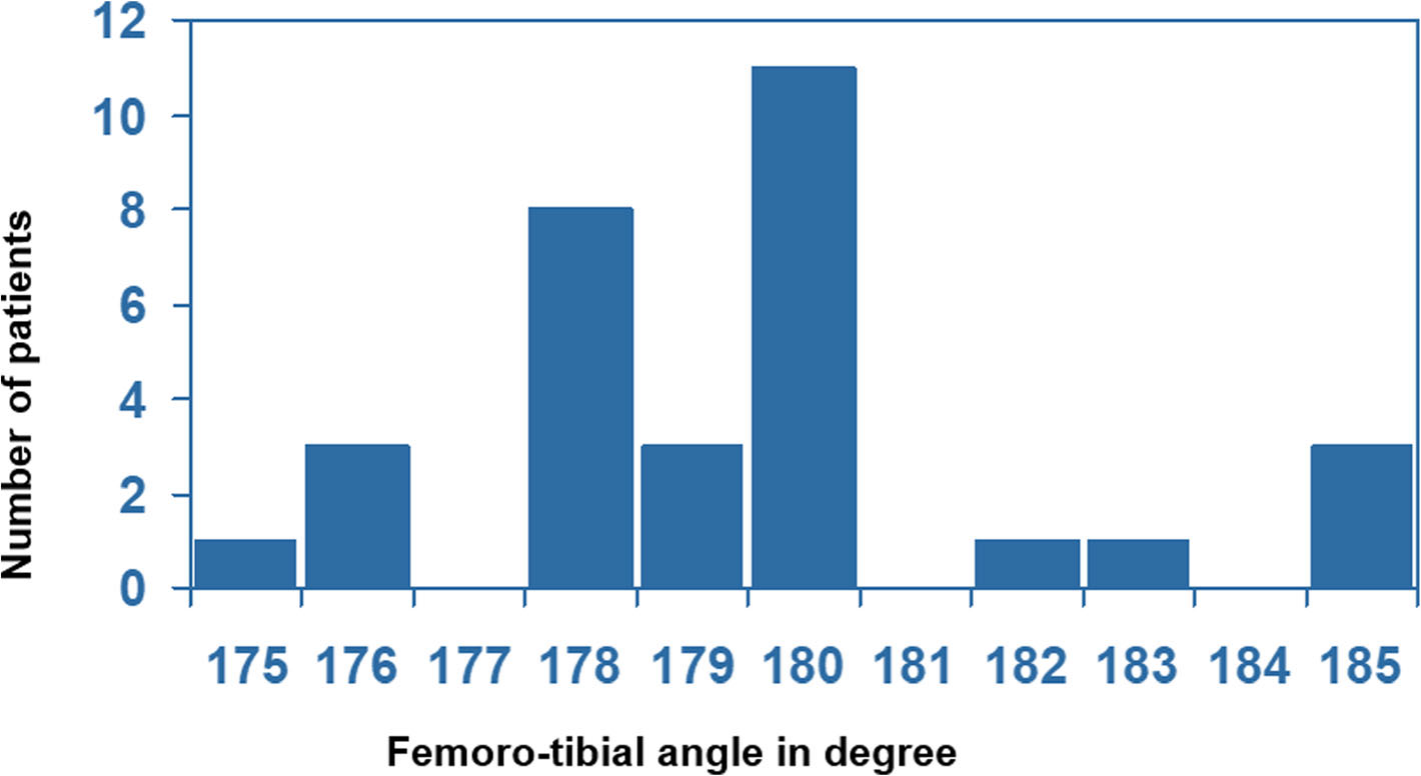
Postoperative mechanical leg axis alignment

We recorded a correction of the mechanical axis in long-leg views from 4° of varus (SD 2.3) preoperatively to a mean varus of 0.6° (SD 2.5). We aimed for a slight under-correction of the mechanical axis of 0–2° varus. In 22/31 knees the aimed range of 0–2° of varus was achieved. The malalignment rate in total was 29% (4 knees were under-corrected with up to 5° of varus, 5 knees were over-corrected to a valgus alignment of less than 5°) (Fig. 3). There was no relationship between malalignment (2–5° varus or valgus) and reduced outcome of the FJS.

## Discussion

Main finding of this study is that CM-UKA for unicondylar OA of the knee can provide improved clinical and functional outcome. This is the first study to report on PRO of custom-made implants of a UKA. We found good results regarding patient satisfaction indicated by a low level of postoperative joint awareness measured by the FJS. These findings are also reflected in good short-term clinical and radiological results at an average follow-up of 2.4 years.

UKA is a well-established procedure for treatment of unicondylar OA of the knee. However, the clinical and radiological results are heterogeneous. There is an ongoing discussion about criteria for patient selection and improvement of surgical technique to optimize postoperative outcome [31–33]. With a mean age of 67 years, our patient population had the typical age distribution in patients requiring UKA for unicondylar compartment OA. The BMI had no significant effect on postoperative outcome, consistent with findungs in previous studies by Murray et al. [34] and Thompson et al. [35]

Prior studies have shown that surgeon-related factors like the implantation technique are of great importance since inadequate positioning of the implant is the main cause for early failure [36]. Generally, implantation of UKA is technically more challenging than TKA, especially with regards to implant alignment and placement [37]. Overcorrection of the leg axis can lead to progression of OA with persistent pain in the contralateral compartment, and should therefore be avoided [18]. Severe under-correction (HKA angle < 170°) is equally undesirable due to wear of the inlay [38]. Hernigou et al. even suggested that a varus alignment with a HKA angle of up to 171° leads to lower wear [36]. Koeck et al. described the target of leg axis correction with this specific implant as a slight under-correction of 0–2° of varus [17, 18]. In our patient population, we were able to achieve this target in 22 of the 31 cases. Mullaji et al. report that a preoperatively strongly deviating leg axis was more likely to present a postoperative remaining varus, whereas a leg axis with only a slight varus was more likely to achieve the wanted 0–2° of varus [39]. Gulati et al. state that malalignment does not allow conclusions about the level of patient satisfaction [40]. Our results on the FJS support this assumption. However, this refers to patients with a moderate postoperative deviation of axis less than 5° [41].

Mechanical malalignment of the implant is seen as a risk factor for early loosening. Batailler et al. recently published a study on robotic-assisted UKA compared to a conventional free-hand implantation of UKA. They found a decreased misalignment rate of a postoperative leg axis deviation of more than 2° in 16% of robotic-assisted UKA, compared to 32% in the control group treated with conventional free-hand implantation. We found a malalignment rate of 29% and no case of a varus or valgus deviation greater than 5° in our population. Batailler et al. recorded patients with a malalignment of more than 10°, even in the robotic-assisted group. In their study, malalignment was the reason for revision in three patients of the control group and they observed three revisions for aseptic loosening after a mean follow-up of 19.7 months. We did not record any revision due to malalignment or aseptic loosening in our population. However, long-term studies are required to analyze survivorship of this type of implant.

Several studies have proven superior functional abilities and patient satisfaction for a UKA compared to TKA [2, 41–46]. It should be noted that there is still a significant number of dissatisfied patients even among those with an excellent outcome based on objectifiable clinical and radiological criteria [38, 47]. The influence of PRO in clinical decision making is rising. Several authors have investigated PRO during the first 2 years after knee arthroplasty and found no relevant change later than 12 months postoperatively [26, 48, 49]. Accordingly, we chose a minimum-follow-up of 1 year postoperatively for evaluation in this study.

In comparison to studies with a similar collective and observational period for unicompartmental prosthesis, our results are similar regarding satisfaction, KSS for pain and function, pain reduction and FJS [42, 44]. The postoperative increase of the KSS after CM-UKA exceeded MICD of 7.2 points by far [50]. Reduction of pain, measured by the VAS for pain, was also at a clinically relevant level of 1.6 [51]. The change in the nominal Tegner Activity Scale increased by 0.7 levels. The ultimate goal in UKA should be the complete unawareness oblivion of the knee in patients’ everyday life. But even results of a healthy group of controls reported by Behrend et al. have shown a FJS of 86.6 (SD 17) for male and 79.3 (SD 23.2) for female participants, therefore showing that even healthy subjects become aware of their knee joints in everyday life [19].

Flury et al. examined the outcome of patient specific UKA 55 months after implantation. FJS was 87 (SD 23) and 89.6% of all patients reported satisfaction during the final follow up. They found an implant survival rate of 92% and excellent accuracy regarding component placement in UKA [52]. Even though well-established standard implants result in a lower FJS than patient specific implants, UKA in general shows superior results than TKA.

A study by Zuiderbaan evaluated FJS in standard UKA and found a FJS of 74.3 ± 24.8, compared to a FJS of 59.8 ± 31.5 in TKA 2 years after implantation [53].

Baumann et al. compared the FJS of patients with bicruciate-retaining implants (53.4 ± 26.4), cruciate-sacrificing implants (38.9 ± 22.0) and standard UKA (53.6 ± 22.2) 18 months postoperatively [7]. Stempin et al. conducted a study to evaluate the medium-term outcome of cementless, mobile-bearing, unicompartmental knee arthroplasty and found a FJS of 75.5 (SD 5.5) after 5 years [54]. Peersmann et al. evaluated patients in a prospective cohort study and found UKA patients with a FJS of 91.3 (range 85.3–97.3) to be less aware of their joint replacements than TKA patients with a FJS of 54.8 (range 49.3–60.2) 1 year after implantation [55]. Therefore implant survival rates and patient satisfaction in the present study are comparable to the highest levels reported for standard, non-customized UKA implant models in literature.

Hence the concept of individualizing with a CM UKA may be beneficial for treatment of medial compartmental OA of the knee. However, careful patient selection is crucial to maintaining a high level of satisfaction in UKA.

This study has some limitations. The main limitation is the non-controlled design of the study. Since UKA is a well-established procedure, there are a number of studies on FJS after UKA that were published since we compared our data to literature. From a scientific point of view, a postoperative evaluation by CT would have provided greater accuracy in radiographic measurements. But since this is a clinical study involving patients, this would not have been compliant with the Ethics Committee’s requirements for patient safety. Additionally, follow-up spans a long period of time for logistic reasons. The procedure of custom-made implants and instruments is time-consuming and costly. Therefore, the recruitment period was 2 years, affecting the range of follow-up as well. Lastly, the follow-up period does not allow a conclusion on the long-term results. Further studies are needed to investigate if the durability of CM-UKA.

## Conclusion

Custom-made unicondylar knee arthroplasty (CM-UKA) can provide improved clinical and functional outcome for patients with isolated knee OA of the medial compartment. This is the first study to report on PRO of CM-UKA. We found excellent results regarding patient satisfaction indicated by a reduced level of joint awareness. Further studies are needed to investigate long-term survivorship of CM-UKA implants.

## Data Availability

Please contact the corresponding author for data requests.

## Abbreviations

CM-UKA: Costum-made unicondylar knee arthroplasty
FJS: Forgotten Joint Score
HR-QoL: Health-related quality of life
KLS: Kellgren and Lawrence Score
KSS: Knee Society Score
MICD: Minimal Important Clinical Difference
OA: Osteoarthritis
PRO: Patient-reported outcome
ROM: Range of motion
TKA: Total knee arthroplasty
VAS: Visual Analogue Scale

## References

[1] Marmor L (1988). Unicompartmental arthroplasty of the knee with a minimum ten-year follow-up period. Clin Orthop Relat Res.

[2] O’Rourke MR, Gardner JJ, Callaghan JJ, Liu SS, Goetz DD, Vittetoe DA (2005). The John Insall award: unicompartmental knee replacement: a minimum twenty-one-year followup, end-result study. Clin Orthop Relat Res.

[3] Spahn G, Hofmann GO, von Engelhardt LV, Li M, Neubauer H, Klinger HM (2013). The impact of a high tibial valgus osteotomy and unicondylar medial arthroplasty on the treatment for knee osteoarthritis: a meta-analysis. Knee Surg Sports Traumatol Arthrosc.

[4] Ji JH, Park SE, Song IS, Kang H, Ha JY, Jeong JJ (2014). Complications of medial Unicompartmental knee Arthroplasty. Clin Orthop Surg.

[5] Koskinen E, Paavolainen P, Eskelinen A, Pulkkinen P, Remes V (2007). Unicondylar knee replacement for primary osteoarthritis: a prospective follow-up study of 1,819 patients from the Finnish Arthroplasty register. Acta Orthop.

[6] Liddle AD, Judge A, Pandit H, Murray DW (2014). Adverse outcomes after total and unicompartmental knee replacement in 101 330 matched patients: a study of data from the National Joint Registry for England and Wales. Lancet.

[7] Baumann F, Krutsch W, Worlicek M, Kerschbaum M, Zellner J, Schmitz P (2018). Reduced joint-awareness in bicruciate-retaining total knee arthroplasty compared to cruciate-sacrificing total knee arthroplasty. Arch Orthop Trauma Surg.

[8] Rougraff BT, Heck DA, Gibson AE (1991). A comparison of tricompartmental and unicompartmental arthroplasty for the treatment of gonarthrosis. Clin Orthop Relat Res.

[9] Liddle AD, Pandit H, Judge A, Murray DW (2016). Effect of surgical caseload on revision rate following Total and Unicompartmental knee replacement. J Bone Joint Surg Am.

[10] Baker P, Jameson S, Critchley R, Reed M, Gregg P, Deehan D (2013). Center and surgeon volume influence the revision rate following unicondylar knee replacement: an analysis of 23,400 medial cemented unicondylar knee replacements. J Bone Joint Surg Am.

[11] Hirschmann MT, Behrend H (2018). Functional knee phenotypes: a call for a more personalised and individualised approach to total knee arthroplasty?. Knee Surg Sports Traumatol Arthrosc.

[12] Jones GG, Logishetty K, Clarke S, Collins R, Jaere M, Harris S (2018). Do patient-specific instruments (PSI) for UKA allow non-expert surgeons to achieve the same saw cut accuracy as expert surgeons?. Arch Orthop Trauma Surg.

[13] Carpenter DP, Holmberg RR, Quartulli MJ, Barnes CL (2014). Tibial plateau coverage in UKA: a comparison of patient specific and off-the-shelf implants. J Arthroplast.

[14] Tegner Y, Lysholm J (1985). Rating systems in the evaluation of knee ligament injuries. Clin Orthop Relat Res.

[15] Insall JN, Dorr LD, Scott RD, Scott WN (1989). Rationale of the knee society clinical rating system. Clin Orthop Relat Res.

[16] Kozinn SC, Scott R (1989). Unicondylar knee arthroplasty. J Bone Joint Surg Am.

[17] Koeck FX, Beckmann J, Luring C, Rath B, Grifka J, Basad E (2011). Evaluation of implant position and knee alignment after patient-specific unicompartmental knee arthroplasty. Knee..

[18] Ridgeway SR, McAuley JP, Ammeen DJ, Engh GA (2002). The effect of alignment of the knee on the outcome of unicompartmental knee replacement. J Bone Joint Surg (Br).

[19] Behrend H, Giesinger K, Giesinger JM, Kuster MS (2012). The ‘forgotten joint’ as the ultimate goal in joint arthroplasty: validation of a new patient-reported outcome measure. J Arthroplast.

[20] Shelburne KB, Kim H-J, Sterett WI, Pandy MG (2011). Effect of posterior tibial slope on knee biomechanics during functional activity. J Orthop Res.

[21] Wewers ME, Lowe NK (1990). A critical review of visual analogue scales in the measurement of clinical phenomena. Res Nurs Health.

[22] Ingelsrud LH, Roos EM, Terluin B, Gromov K, Husted H, Troelsen A (2018). Minimal important change values for the Oxford knee score and the forgotten joint score at 1 year after total knee replacement. Acta Orthop.

[23] Baumann F, Ernstberger T, Loibl M, Zeman F, Nerlich M, Tibesku C (2016). Validation of the German forgotten joint score (G-FJS) according to the COSMIN checklist: does a reduction in joint awareness indicate clinical improvement after arthroplasty of the knee?. Arch Orthop Trauma Surg.

[24] Shadid MB, Vinken NS, Marting LN, Wolterbeek N (2016). The Dutch version of the forgotten joint score: test-retesting reliability and validation. Acta Orthop Belg.

[25] Thienpont E, Opsomer G, Koninckx A, Houssiau F (2014). Joint awareness in different types of knee arthroplasty evaluated with the forgotten joint score. J Arthroplast.

[26] Thomsen MG, Latifi R, Kallemose T, Husted H, Troelsen A (2016). Does knee awareness differ between different knee arthroplasty prostheses? A matched, case-control, cross-sectional study. BMC Musculoskelet Disord.

[27] Kellgren JH, Lawrence JS (1957). Radiological assessment of osteo-arthrosis. Ann Rheum Dis.

[28] Sperner G, Wanitschek P, Benedetto KP, Glötzer W (1990). Late results in patellar fracture. Aktuelle Traumatol.

[29] Gulati A, Chau R, Pandit HG, Gray H, Price AJ, Dodd C a F (2009). The incidence of physiological radiolucency following Oxford unicompartmental knee replacement and its relationship to outcome. J Bone Joint Surg (Br).

[30] Doberstein ST, Romeyn RL, Reineke DM (2008). The diagnostic value of the Clarke sign in assessing chondromalacia patella. J Athl Train.

[31] Kasodekar VB, Yeo SJ, Othman S (2006). Clinical outcome of unicompartmental knee arthroplasty and influence of alignment on prosthesis survival rate. Singap Med J.

[32] Skowroński J, Jatskewych J, Długosz J, Skowroński R, Bielecki M (2005). The Oxford II medial unicompartmental knee replacement. A minimum 10-year follow-up study. Ortop Traumatol Rehabil.

[33] Stern SH, Becker MW, Insall JN (1993). Unicondylar knee arthroplasty. An evaluation of selection criteria. Clin Orthop Relat Res.

[34] Murray DW, Pandit H, Weston-Simons JS, Jenkins C, Gill HS, Lombardi AV (2013). Does body mass index affect the outcome of unicompartmental knee replacement?. Knee..

[35] Thompson SAJ, Liabaud B, Nellans KW, Geller JA (2013). Factors associated with poor outcomes following unicompartmental knee arthroplasty: redefining the ‘classic’ indications for surgery. J Arthroplast.

[36] Hernigou P, Deschamps G (2004). Alignment influences wear in the knee after medial unicompartmental arthroplasty. Clin Orthop Relat Res.

[37] Markel DC, Sutton K (2005). Unicompartmental knee arthroplasty: troubleshooting implant positioning and technical failures. J Knee Surg.

[38] Sarangi PP, Karachalios T, Jackson M, Newman JH (1994). Patterns of failed internal unicompartmental knee prostheses, allowing persistence of undercorrection. Rev Chir Orthop Reparatrice Appar Mot.

[39] Mullaji AB, Shetty GM, Kanna R (2011). Postoperative limb alignment and its determinants after minimally invasive Oxford medial unicompartmental knee arthroplasty. J Arthroplast.

[40] Gulati A, Pandit H, Jenkins C, Chau R, Dodd C (2009). a. F, Murray DW. The effect of leg alignment on the outcome of unicompartmental knee replacement. J Bone Joint Surg (Br).

[41] Kim KT, Lee S, Kim TW, Lee JS, Boo KH (2012). The influence of postoperative Tibiofemoral alignment on the clinical results of Unicompartmental knee Arthroplasty. Knee Surg Relat Res.

[42] Kehr P, Nonn P, Graftiaux A, Bogorin I, Leculée F, Lang G (1995). The ‘Oxford’ unicondylar knee prostesis (UCP): 21 reviewed cases. Eur J Orthop Surg Traumatol.

[43] Riddle DL, Jiranek WA, McGlynn FJ (2008). Yearly incidence of unicompartmental knee arthroplasty in the United States. J Arthroplast.

[44] Parmaksizoğlu AS, Kabukçuoğlu Y, Ozkaya U, Bilgili F, Aslan A (2010). Short-term results of the Oxford phase 3 unicompartmental knee arthroplasty for medial arthritis. Acta Orthop Traumatol Turc.

[45] Arirachakaran A, Choowit P, Putananon C, Muangsiri S, Kongtharvonskul J (2015). Is unicompartmental knee arthroplasty (UKA) superior to total knee arthroplasty (TKA)? A systematic review and meta-analysis of randomized controlled trial. Eur J Orthop Surg Traumatol.

[46] Ettinger M, Zoch JM, Becher C, Hurschler C, Stukenborg-Colsman C, Claassen L (2015). In vitro kinematics of fixed versus mobile bearing in unicondylar knee arthroplasty. Arch Orthop Trauma Surg.

[47] Fehring TK, Odum SM, Masonis JL, Springer BD (2010). Early failures in unicondylar arthroplasty. Orthopedics..

[48] Fitzgerald JD, Orav EJ, Lee TH, Marcantonio ER, Poss R, Goldman L (2004). Patient quality of life during the 12 months following joint replacement surgery. Arthritis Rheum.

[49] Pynsent PB, Adams DJ, Disney SP (2005). The Oxford hip and knee outcome questionnaires for arthroplasty. J Bone Joint Surg (Br).

[50] Lizaur-Utrilla A, Gonzalez-Parreño S, Martinez-Mendez D, Miralles-Muñoz FA, Lopez-Prats FA (2020). Minimal clinically important differences and substantial clinical benefits for knee society scores. Knee Surg Sports Traumatol Arthrosc.

[51] Danoff JR, Goel R, Sutton R, Maltenfort MG, Austin MS (2018). How much pain is significant? Defining the minimal clinically important difference for the visual analog scale for pain after Total joint Arthroplasty. J Arthroplast.

[52] Flury A, Hasler J, Dimitriou D, Antoniadis A, Finsterwald M, Helmy N (2019). Midterm clinical and radiographic outcomes of 115 consecutive patient-specific unicompartmental knee arthroplasties. Knee..

[53] Zuiderbaan HA, van der List JP, Khamaisy S, Nawabi DH, Thein R, Ishmael C (2017). Unicompartmental knee arthroplasty versus total knee arthroplasty: which type of artificial joint do patients forget?. Knee Surg Sports Traumatol Arthrosc.

[54] Stempin R, Stempin K, Kaczmarek W (2019). Medium-term outcome of cementless, mobile-bearing, unicompartmental knee arthroplasty. Ann Transl Med.

[55] Peersman G, Verhaegen J, Favier B (2019). The forgotten joint score in total and unicompartmental knee arthroplasty: a prospective cohort study. Int Orthop.

